# Analysis of Morphokinetic Parameters of Feline Embryos Using a Time-Lapse System

**DOI:** 10.3390/ani11030748

**Published:** 2021-03-09

**Authors:** Joanna Kochan, Agnieszka Nowak, Barbara Kij, Sylwia Prochowska, Wojciech Niżański

**Affiliations:** 1 Department of Animal Reproduction, Anatomy and Genomics, University of Agriculture, Mickiewicza 24/28, 30-059 Krakow, Poland; nowak.a.a@gmail.com (A.N.); barbara.kij@op.pl (B.K.); 2 Department of Reproduction and Clinic of Farm Animals, University of Environmental Science, Grundwaldzki Square 49, 50-357 Wroclaw, Poland; sylwia.prochowska@upwr.edu.pl (S.P.); wojciech.nizanski@upwr.edu.pl (W.N.)

**Keywords:** felids, embryos, IVF, time-lapse, morphokinetic

## Abstract

**Simple Summary:**

This study was conducted with the aim of analyzing the morphokinetic parameters that determine the proper development of feline embryos in vitro. Our research was carried out using a time-lapse monitoring system shows that the timing of the first and second cleavage divisions, the timing of blastocyst cavity formation and morphological anomalies can all be used as early and non-invasive indicators of cat embryo development in vitro.

**Abstract:**

The aim of this study was to analyze the morphokinetic parameters of feline embryos using a time lapse system. Oocytes matured in vitro were fertilized (IVF) and in vitro cultured in a time lapse-system (Primo Vision^®^, Gothenburg, Sweden). The first cell division of embryos occurred between 17 h post insemination (hpi) and 38 hpi, with the highest proportion of embryos (46%) cleaving between 21 and 24 hpi. The timing of the first cleavage significantly affected further embryo development, with the highest development occurring in embryos that cleaved at 21–22 hpi. Embryos that cleaved very early (17–18 hpi) developed poorly to the blastocyst stage (2%) and none of the embryos that cleaved later than 27 hpi were able to reach the blastocyst stage. Morphological defects were observed in 48% of the embryos. There were no statistically significant differences between the timing intervals of the first cleavage division and the frequency of morphological defects in embryos. Multiple (MUL) morphological defects were detected in more than half (56%) of the abnormal embryos. The most frequent single morphological defects were cytoplasmic fragmentation (FR) (8%) and blastomere asymmetry (AS) (6%). Direct cleavage (DC) from 1–3 or 3–5 blastomeres, reverse cleavage (RC) and vacuoles were rarely observed (2–3%). The timing of blastocyst cavity formation is a very good indicator of embryo quality. In our study, blastocyst cavity formation occurred between 127–167 hpi, with the highest frequency of hatching observed in blastocysts that cavitated between 142–150 hpi. Blastocysts in which cavitation began after 161 h did not hatch. In conclusion, the timing of the first and second cleavage divisions, the timing of blastocyst cavity formation and morphological anomalies can all be used as early and non-invasive indicators of cat embryo development in vitro.

## 1. Introduction

The domestic cat (*Felis catus*) is frequently used in research as a model for other feline species threatened with extinction. Many assisted reproduction techniques such as artificial insemination, in vitro fertilization (IVF), intracytoplasmic sperm injection (ICSI) and somatic cell cloning (NT) are tested using the domestic cat as the most available source of research material in comparison to wild felids [1,2,3].

Although the first domestic cat kittens derived as a result of in vitro fertilization (IFV) were born 30 years ago [4], production efficiency remains low compared with other animal species, as an average of only 50% of inseminated cat oocytes develop into cleaved embryos and only 20% of these embryos reach the blastocyst stage [5,6,7]. Progress in this area will rely on developing a better understanding of the mechanisms of fertilization and optimization of the in vitro culture conditions for feline embryos. Until now, the quality of feline embryos produced in vitro has been determined on the basis of their morphological quality, the numbers of cells in the blastocyst inner cell mass (ICM) and trophectoderm (TE), the incidence of apoptosis, and expression of specific genes [8,9,10,11,12]. Since most of these analyses require the destruction of the embryo, the most common non-invasive method used for the assessment of embryo quality and selection for embryo transfer is the standard morphological evaluation.

Time-lapse monitoring is a non-invasive, effective method for the continuous imaging of the development of each individual embryo in vitro, allowing for the analysis of its morphokinetics, blastomere number, symmetry of cell division and the extent of cytoplasmic fragmentation. Time-lapse videorecording during embryo culture provides the maximum amount of information to support the optimal embryo selection while accurately recording all details of embryo development. Moreover, time lapse systems are reliable and objective, which enables the comparison and review of results between different laboratories, even in the case of inexperienced embryologists.

Time-lapse systems have been successfully used for the evaluation and selection of human embryos for transfer [13,14,15], and in research on the development of mouse and bovine embryos [16,17,18]. In many species, the crucial role of first cleavage timing has been emphasized as an additional marker of embryo viability correlated with their developmental and implantation potential [16,19,20]. In cats, the association between the onset of the first cleavage division and blastocyst formation has only been studied using traditional microscopic observation of embryos at 18, 24 and 30 h post-insemination [12] or 27 and 42 h post-insemination [21]. In our previous pilot studies [22], we used time lapse to monitor feline embryos for the first time and determined the frequency of morphological defects of embryos and their competence to reach the blastocyst stage, as well as their ability to hatch. Now, as a continuation of that research, we have made an attempt to precisely define morphokinetic parameters in feline embryos using a time lapse system (Primo Vision^®^).

## 2. Materials and Methods

### 2.1. Source of Ovaries and Oocyte Collection

Ovaries and oocytes were collected according to the procedure described by Kochan et al. [23]. Ovaries were collected after ovariohysterectomy from healthy adult domestic cats (*n* = 124) at local veterinary clinics. The fertility history of these cats was not known. Ovaries were kept in Dulbecco’s phosphate buffered saline (DPBS) supplemented with 100 μg/mL of streptomycin and 100 IU/mL of penicillin at 4 °C in a thermally insulated box. Oocytes were obtained within 1–3 h after ovariohysterectomy.

Cumulus-oocyte complexes (COCs) were obtained by scarification of the ovarian cortex in a washing medium (TCM 199 with Earle’s salts, HEPES-buffered and supplemented with 3 mg/mL BSA, 0.1 mg/mL cysteine, 0.25 mg/mL sodium pyruvate, 0.6 mg/mL sodium lactate, 0.15 mg/mL L-glutamine and 0.055 mg/mL gentamycin).

### 2.2. In Vitro Maturation (IVM) of Oocytes

Only oocytes with uniform and dark cytoplasm, surrounded by compact layers of cumulus cells, were selected for IVM. Selected COCs were placed into 400 µL of maturation medium, described above as a washing medium, supplemented with 0.02 IU FSH/mL and 0.02 IU LH/mL under mineral oil and cultured for 24 h at 38.5 °C underX 5% CO_2_ in air.

### 2.3. In Vitro Fertilization (IVF)

Oocytes surrounded by several layers of expanded cumulus cells were used for in vitro fertilization (IFV). Thawed spermatozoa isolated from cauda epididymis and frozen according to theprocedure described by Niżański et al. [24] were used. Before IVF, semen was thawed at 37 °C for 30 s and motile spermatozoa were selected by swim-up processing in Sperm Air^®^ medium (Gynemed, Lensahn, Germany). Groups of oocytes were inseminated with 5 × 10^5^ motile spermatozoa/mL in 50-μL microdrops of CULT^®^ Medium (Gynemed, Lensahn, Germany) under mineral oil at 38.5 °C in air with 5% CO_2_ and cultured for 16 h. Oocytes were then transferred to the time-lapse system to capture the time of first division of embryos.

### 2.4. Embryo Culture in the Time-Lapse System (Primo Vision)

A 16-well dish was prepared and equilibrated in the incubator for 2 h before embryo culture. Individual wells were filled with culture medium CULT^®^ (Gynemed, Germany) and then covered with a common drop of 60 μL of medium CULT^®^ (Gynemed, Germany), after which the whole dish was covered with mineral oil (4 mL). Then, the embryos were placed into the microwells. The dish, with individually identified microwells for each embryo, was placed in the dish holder of the Primo Vision^®^ Digital Microscope (Vitrolife, Gothenburg, Sweden) inside the incubator. The Digital Microscope takes images of embryos during their in vitro development at 5 min frequencies. The morphokinetic parameters were determined by an operator looking at the recorded images. The medium was partially changed (30 μL) every other day. Any embryos that had arrested division were removed from the dish. The in vitro culture was finished when blastocysts hatched or if they did not start hatching by the end of the 8th day (192 h hpi).

### 2.5. Embryo Evaluation

Morphokinetic parameters were determined for all embryos:

- t2, t3, t4—The timing of division to the 2, 3 and 4 blastomere stage
- BC—formation of the blastocyst cavity
- BL—expanded blastocyst
- BH—hatching blastocyst
- N—normal
- AS—asymmetry of blastomeres
- FR—cellular fragmentation
- DC—direct cleavage from one to three or more blastomeres
- RC—reverse cleavage (blastomere fusion after division)
- VAC—vacuoles in blastomeres
- MUL—presence of two or more morphological defects.
- Proportions of late embryo stages (blastocyst, hatching blastocysts) were calculated as a function of the total number of cleaved embryos.

### 2.6. Statistical Analysis

The relationship between cleavage time and subsequent embryo development, and the influence of time of blastocyst cavitation to blastocyst hatching were tested using Fisher’s exact test depending on the number of variants of analyzed variables. The level of statistical significance was set at *p* < 0.05. The statistical analysis was performed using PQStat 1.6.2 for Windows 2016 (PQStat Soft, Poznan, Poland).

## 3. Results

**Figure 1:**
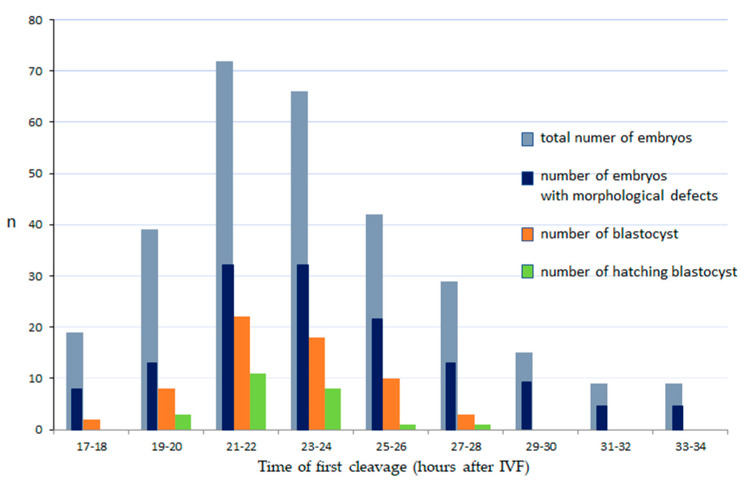
The relationship between the time of first cleavage, incidence of morphological defects and development of domestic cat embryos. IVF: in vitro fertilization. Number of cleaved embryos, blastocyst and hatching blastocysts: there were statistically significant differences between those with first cleavage at 21–24 hpi and other time intervals (*p* < 0.001).

A total of 1460 COCs were collected in 33 repetitions of the experiment, of which 805 were suitable for fertilization (IVF). Of 805 oocytes inseminated, 300 reached the cleavage stage (37%); and of these 153 (51%) developed to the morula stage, 63 (21%) reached the blastocyst stage and 24 (8%) hatched. The earliest divisions of embryos were observed at 17 hpi and the latest at 38 hpi. The highest proportion of embryos cleaved between 21 and 24 hpi (46%, *p* < 0.001); (Figure 1, Video S4).

The timing of the first cleavage significantly predicted further embryo development (Figure 1.). Embryos that cleaved at 21–24 hpi had the highest development potential (*p* < 0.001), while those that cleaved very early (17–18 hpi) had poor development to the blastocyst stage (2%) and none that cleaved later than 27 hpi were able to reach the blastocyst stage.

**Figure 2:**
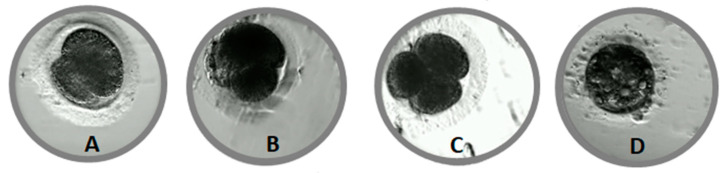
Morphological defects in cat embryos. (**A**)—asymmetry of blastomeres, (**B**)—cellular fragmentation, (**C**)—direct cleavage, (**D**)—vacuoles.

Morphological defects were observed in 144 (48%) embryos. There was no relationship between the time of the first cleavage and the incidence of morphological defects in embryo. Multiple (MUL) morphological defects were detected in more than half (56%) of the abnormal embryos (Table 1). The most frequent single morphological defects were cytoplasmic fragmentation (FR, Figure 2B) (8%) and blastomere asymmetry (AS, Figure 2A), (6%). Direct cleavage (DC, Figure 2C, Video S3) from 1–3 or 3–5 blastomeres, reverse cleavage (RC) and vacuoles (Figure 2D) were rarely observed (2–3%).

**Figure 3:**
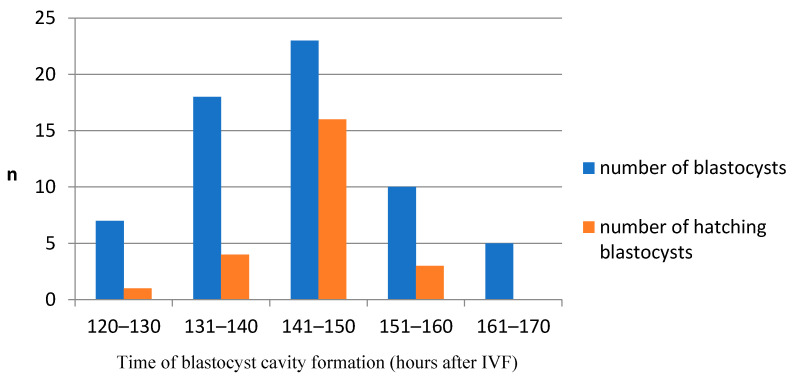
The relationship between the time of cavity formation and the hatching ability in domestic cat blastocysts. Blastocysts that formed between 141–150 hpi had a significantly higher incidence of hatching (*p* < 0.001).

**Figure 4:**
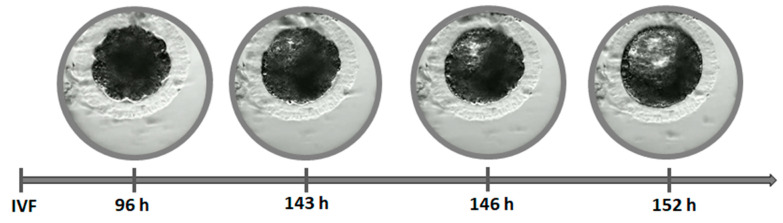
Timing of cavitation and blastocyst formation.

**Figure 5:**
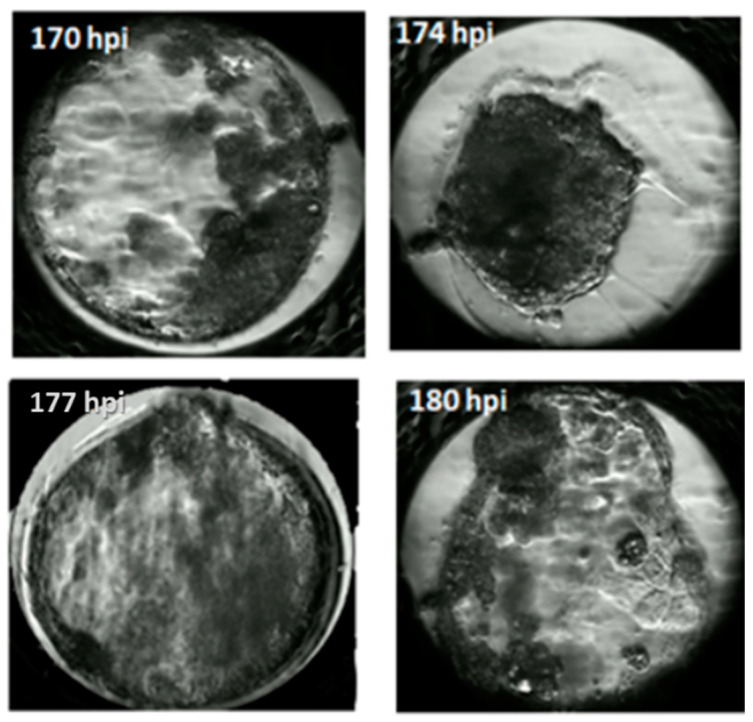
Timing of blastocyst hatching.

In our study, the blastocyst cavity was formed between 127 hpi and 167 hpi (Figure 3, Video S1). The cavity in feline blastocyst is quite evident and the timing of cavity formation is easy to determine (Figure 4). The highest frequency of hatching was noted in blastocysts that formed between 141–150 hpi (*p* < 0.001). Figure 5 shows the process of the hatching of a randomly selected blastocyst (Video S2). Blastocysts in which cavitation began after 161 hpi did not hatch.

## 4. Discussion

Recently, many studies have indicated that the timing of the first embryonic cleavage is a simple and noninvasive method that can be used as an early indicator for selecting good quality embryos, as shown in cattle [25], humans [26] and sheep [20,27]. In some studies, to eliminate error associated with variation in the time of sperm entry, the timing of pronuclear fading (tPNF) was proposed as a reference starting time [28]. In the case of cats, it is not possible to assess the formation or disappearance of pronuclei in oocytes because of their dark cytoplasm. In our study (Figure 1), feline embryos that cleaved relatively early (21–24 hpi) were more developmentally competent compared with those that cleaved later (>25 hpi) (*p* < 0.001). Although the earliest initiation of first cleavage was observed at 17 hpi, these embryos had a very poor rate of development to the blastocyst stage (2%). These results are in accordance with previous studies in several species such as cattle [16,19,20,29]. In cats, the relationship between the timing of the first cleavage on embryo developmental competence has not been examined using a time-lapse system, only via traditional observation of embryonic development at 18,24 and 30 hpi [12], or 27 and 42 hpi [21]. Ochota and co-workers [12] reported that the best quality embryos cleaved before 18–24 hpi, while none of the embryos that cleaved between >24–30 hpi reached the blastocyst stage. This is different from our data and from the data reported by Klincumhom and co-workers [21] who found blastocyst formation in embryos that cleaved between 27 and 42 hpi. These discrepancies from our results may be due to Ochota et al. [12] and Klincumhom et al. [21] using only three time points for the assessment of the first cleavage of feline embryos, and they ended their observations at 30 or 42 hpi. The analysis of embryo morphokinetics allows for much more precise, continuous observations. In our study, using time-lapse monitoring, the latest incidence of first cleavage was recorded at 39 hpi, but blastocysts did not develop from embryos that cleaved later than 27 hpi. In humans, two-cell embryos that cleave at 25–28 hpi are usually selected for transfer to improve the chance of achieving pregnancy [30,31,32].

We observed a large synchronization of the first division for early embryos (22–23 hpi) and the majority of the best quality embryos cleaved at 22(±39 min) hpi. In humans, it was found that the blastocyst formation rate was significantly increased in the high-synchrony (first division synchrony ±3.96 h) compared with the low-synchrony groups (first division synchrony longer than 3.96 h) [31]. Further cell divisions were not synchronous and embryos dividing t2–t3 at 8–15 h after the first cleavage had the highest development rates [31]. In humans, it has been established that the duration of the cell cycle is around 10 to 12 h [32]. This interval is sufficient for the embryo to undergo two consecutive phases of cytokinesis and to replicate the entire cell genome. This was confirmed by Rubio and co-workers [33], who showed that embryos cleaving from two to three cells in less than 5 h had a significantly lower implantation ability than embryos with a normal cell cycle length. Additionally, in our study, embryos that divided quickly and reached the morula stage on day 3 did not develop to the blastocyst stage. Using the time-lapse monitoring system, we detected an abnormally short cell cycle of as little as 3 h. Extremely short cell cycles could be related to other factors and result in incomplete DNA replication, which might then be associated with an unequal distribution of DNA among blastomeres.

Due to the very dark cytoplasm of feline embryos, it is difficult to make an exact determination of the number of blastomeres in subsequent divisions and the time of morula formation. However, it also means that the blastocyst cavity is quite evident, and the timing of cavity formation is a very good indicator of embryo quality (Figure 4). In our study, the blastocyst cavity was formed between 127 hpi and 167 hpi (Figure 3).

In our previous study we determined the incidence of morphological defects in feline embryos [22]. We divided defects into two groups: single or multiple aberration. In the present study, we noticed a similar proportion of abnormal embryos (48 vs. 46%) and of multiple aberrations (27 vs. 32%) compared to the previous experiment, despite the use of different culture media. In the previous study, we used commercial bovine culture media (BO-IVC, Bioscience, Poland) and in the current one commercial human media (CULT, Gynemed, Germany). Moreover, in this study we classified the single defects as: asymmetry of blastomeres (AS), cellular fragmentation (FR), direct cleavage (DC), reverse cleavage (RC), and vacuoles (VAC), (Table 1, Figure 2).

The most notable single defects were cytoplasmic fragmentation and blastomere asymmetry. A high degree of fragmentation can lead to the loss of cytoplasmic organelles, such as mitochondria [34], and further induces necrotic effects in the surrounding blastomeres [35], which causes developmental arrest or low embryo development. However, in our study we did not observe embryos with a high degree of cytoplasmic fragmentation (>50%). Rarely observed morphological defects in feline embryos were direct cleavage (DC) (3%) or reverse cleavage (RC) (2%). Direct cleavage significantly reduced the ability of embryos to reach the blastocyst stage. After reverse cleavage, no blastocysts were observed, but this occurred so rarely (three embryos) that it is impossible to objectively determine the significance of the impact of this defect on blastocyst development. The causes of DC or RC remain unknown. In humans, DC is believed to be related to the formation of multipolar spindles, which cause the abnormal segregation of chromosomes during cleavage [36]. Moreover, embryos with DC have a higher likelihood of an abnormal ploidy status or chromosome number abnormalities [37]. One study reported that RC is associated with aneuploidy by using array comparative genomic hybridization (array-CGH) [38]. Morphological defects such as DC and RC are only visible for a short time and may go unnoticed in traditional microscopic evaluation performed every 24 h.

The rarest morphological defect was the appearance of vacuoles in the blastomeres, which was recorded in only one embryo. This embryo developed to the blastocyst stage but was of very poor quality. Vacuoles in embryos are a consequence of vacuoles already present in oocytes. Vacuoles are membrane-bound cytoplasmic inclusions filled with a fluid that is virtually identical to perivitelline fluid. They vary in size as well as in number and can be observed in 5–12% of human oocytes [39]. It has been shown that vacuolated human oocytes exhibit significantly reduced fertilization rates and developmental ability [40]. However, healthy offspring derived from such embryos have also been reported [41]. So far, there are no data on the occurrence of vacuoles in feline embryos.

There were no statistically significant differences between the timing intervals of the first cleavage division and the incidence of morphological defects in embryos.

According to our previous research [22], embryos with morphological disorders have the potential to reach the blastocyst stage as well as normal embryos but are less likely to hatch. The hatching rate was the highest in the normally cleaving embryos (15.6%) and decreased significantly within the groups that exhibited a single aberration (6.25%) and multiple aberrations (3.33%).

## 5. Conclusions

In this pioneer study, we have established precisely the morphokinetics of feline embryos. The timing of the first and second cleavage divisions, the timing of blastocyst cavity formation and morphological anomalies can all be used to select good quality embryos and may be used as early and non-invasive indicators of in vitro development of normal cat embryos.

## Figures and Tables

**Table 1:** Characterization of morphological defects in domestic cat embryos.

Morphology of Embryos	N (%)
Total number of embryos	300 (100)
Normal	156 (52)
Abnormal	144 (48)
MUL—multiple defects	81 (27)
FGR—cellular fragmentation	24 (8)
AS—asymmetry of blastomeres	18 (6)
DC—direct cleavage	9 (3)
RC—reverse cleavage	6 (2)
VAC—vacuoles in blastomeres	6 (2)

## Data Availability

Data is contained within the article and supplementary material.

## Supplementary Materials

The following are available online at https://www.mdpi.com/2076-2615/11/3/748/s1, Video S1: Blastocyst cavitation, Video S2: Blastocyst hatching, Video S3: DC–direct cleavage of embryo, Video S4: First cleavage; t2, t3, t4.
